# Zinc Oxide and Silver Nanoparticle Effects on Intestinal Bacteria

**DOI:** 10.3390/ma14102489

**Published:** 2021-05-12

**Authors:** Ami Yoo, Mengshi Lin, Azlin Mustapha

**Affiliations:** Food Science Program, Division of Food Systems and Bioengineering, University of Missouri, Columbia, MO 65211, USA; yooami1218@hotmail.com (A.Y.); LinMe@missouri.edu (M.L.)

**Keywords:** antimicrobials, intestinal bacteria, zinc oxide nanoparticles, silver nanoparticles

## Abstract

The application of nanoparticles (NPs) for food safety is increasingly being explored. Zinc oxide (ZnO) and silver (Ag) NPs are inorganic chemicals with antimicrobial and bioactive characteristics and have been widely used in the food industry. However, not much is known about the behavior of these NPs upon ingestion and whether they inhibit natural gut microflora. The objective of this study was to investigate the effects of ZnO and Ag NPs on the intestinal bacteria, namely *Escherichia coli*, *Lactobacillus acidophilus*, and *Bifidobacterium animalis*. Cells were inoculated into tryptic soy broth or Lactobacilli MRS broth containing 1% of NP-free solution, 0, 12, 16, 20 mM of ZnO NPs or 0, 1.8, 2.7, 4.6 mM Ag NPs, and incubated at 37 °C for 24 h. The presence and characterization of the NPs on bacterial cells were investigated by scanning electron microscopy (SEM), transmission electron microscopy (TEM), and energy-dispersive X-ray spectroscopy (EDS). Membrane leakage and cell viability were assessed using a UV-visible spectrophotometer and confocal electron microscope, respectively. Numbers of treated cells were within 1 log CFU/mL less than those of the controls for up to 12 h of incubation. Cellular morphological changes were observed, but many cells remained in normal shapes. Only a small amount of internal cellular contents was leaked due to the NP treatments, and more live than dead cells were observed after exposure to the NPs. Based on these results, we conclude that ZnO and Ag NPs have mild inhibitory effects on intestinal bacteria.

## 1. Introduction

Nanotechnology is widely applied in many industrial fields, such as information and communication technology, biotechnology, pharmaceuticals, computer electronics, medicinal technology, and the food industry [1,2,3,4]. Recently, nanoparticles made of carbon, metal, and metal oxide, have been applied in various areas due to their unique characteristics [2].

Engineered nanoparticles (NPs) are materials that have applications in the food industry, such as in food packaging, to improve the shelf life of food and to prevent microbial contamination [5,6]. Among the metal-based NPs, ZnO NP is one of the most well-studied. ZnO NPs are unique in that they are not only stable under high temperatures and pressures that are typically employed in food processing conditions, but they are also generally regarded as safe (GRAS) [7,8]. The antimicrobial activity of ZnO toward bacteria is dependent on many factors, including the size of the NP, the concentration used, and the type of bacteria targeted (Gram-positive or Gram-negative) [9,10]. Silver (Ag) NPs are inorganic antibacterial agents used in the pharmaceutical and medical industries and have a significant potential for a wide range of biological applications, including as antifungal and antibacterial agents for antibiotic resistant organisms and for preventing infections [11]. Like ZnO NPs, the size and concentration of Ag NPs also directly affect their antimicrobial properties [12,13]. However, the exact mechanisms of effect of ZnO and Ag NPs on bacteria are still unclear.

To improve the properties and reduce the production costs of NPs, many synthetic methods for generating NPs have been developed [14,15]. More recently, the green synthesis method offers a novel and potential alternative to chemically-synthesized NPs [16,17,18]. Our own previous work [19] demonstrated that green-synthesized Ag NPs using turmeric extract showed antimicrobial effect against pathogenic bacteria. Although recent studies have demonstrated the antimicrobial activities of ZnO and Ag NPs toward foodborne pathogenic and spoilage microorganisms [9,11,20,21,22,23,24,25,26,27,28,29], not much is known about the behavior of ZnO and Ag NPs upon ingestion and whether they inhibit natural gut microflora, which are a major part of the human defense system.

In this study, we investigated the effect of ZnO and Ag NPs on three important intestinal bacteria, i.e., *Escherichia coli*, *Lactobacillus acidophilus*, and *Bifidobacterium animalis*. The modes of action of ZnO and Ag NPs on the growth of the bacterial cells were also studied by a combination of microscopic and analytical methods. The results of this study demonstrated that the cytotoxic effects exhibited by ZnO and Ag NPs, at the concentrations and sizes tested, against these intestinal bacteria were minimal.

## 2. Materials and Methods

### 2.1. Preparation of Intestinal Bacterial Strains

*E. coli* K-12, *L. acidophilus* ADH, and *B. animalis* Bif-6 were from the culture collection of the Food Microbiology Laboratory at the University of Missouri, Columbia, MO. *E. coli* K-12 was grown in tryptic soy broth supplemented with 0.5% yeast extract (TSBY; Difco Labs., BD Diagnostics Systems, Sparks, MD, USA). *L. acidophilus* ADH and *B. animalis* Bif-6 were grown in Lactobacilli MRS broth (Difco Labs.) supplemented with 0.05% cysteine. All three strains were freshly prepared in 10 mL of the respective broth medium and tubes were incubated aerobically for *E. coli* and anaerobically for the other two strains for 18–20 h at 37 °C (final cell concentrations of ~10^9^ CFU/mL) [30].

### 2.2. Preparation of Zinc Oxide and Silver Nanoparticles

ZnO NP suspensions with an average particle size of 77 nm were purchased from Alfa Aesar (Ward Hill, MA, USA). The concentration of the original suspension was 12 M. Ag NPs were synthesized by a chemical reduction method according to Ratyakshi and Chauhan (2009) [31]. The size of the ZnO NPs and Ag NPs was determined by analyzing transmission electron microscopy (TEM) images using the ImageJ software available at https://imagej.softonic.kr/download (accessed on 3 April 2021). One hundred different measurements were taken of the ZnO data and 130 different measurements were taken of the Ag NP data [30].

### 2.3. Effect of ZnO and Ag NPs on the Growth of Bacterial Cells

Freshly (overnight) grown cultures of *E. coli* K-12, *L. acidophilus* ADH and *B. animalis* Bif-6 were inoculated into the respective broth medium (TSBY or MRS) containing 0, 12, 16, 20 mM of ZnO NP, 0, 1.8, 2.7, 4.6 mM of Ag NP, or 1% of NP-free solutions. The NP-free solutions were prepared by filtering the respective NP suspensions through an inorganic anodisc membrane with a 20 nm pore size (Whatman Inc., Clifton, NJ, USA). NP suspensions and the NP-free solution were added to the broth media before autoclaving.

Tubes containing *E. coli* K-12 were incubated in a shaker incubator set at 37 °C. Tubes inoculated with *L. acidophilus* ADH and *B. animalis* Bif-6 were first placed in an anaerobic jar with GasPak gas generators (GasPak EZ Anaerobe Container System, BD Diagnostics) before incubating in a shaker incubator set at medium shaking at 37 °C. The reason for the shaking incubation was to avoid aggregation of the NPs in the broth and to allow for a consistent contact between the bacterial cells and the NPs. The samples were diluted with peptone water and plated at varying times for up to 24 h in the respective agar media, *viz*. Tryptic Soy Agar supplemented with yeast extract for *E. coli* K-12, and Lactobacilli MRS agar supplemented with cysteine for *L. acidophilus* ADH and *B. animails* Bif-6. The sampling times between 0 and 12 h differed for experiments with ZnO and those with Ag NPs. In order to capture the most accurate changes in cell numbers, these respective sampling time points were selected based on a preliminary experiment that indicated changes in cell numbers after ZnO exposure occurred after 6 h and those after Ag NP exposure occurred before 6 h [30].

### 2.4. Morphological Examination of the Bacterial Cells

Scanning electron microscopy (SEM) was used to examine the morphological changes of the bacterial cells before and after treating with ZnO NPs and Ag NPs. The three bacterial strains treated or untreated with NPs were fixed with a primary fixative (2.5 glutaraldehyde, 2% paraformaldehyde in 0.1 M Na-cacodylate buffer, pH 7.4). The samples were then rinsed three times with ultrapure water, followed by dehydration with a series of ethanol solutions (10%, 30%, 50%, 70%, 90%, and 100%). The dehydrated samples were immediately dried by a critical point dryer (Auto-Samdri 815 Automatic Critical Point Dryer; Tousimis, Rockville, MD, USA), mounted on SEM stubs and coated with a thin layer of carbon using a sputter coater (K575X Turbo Sputter Coater; Emitech, Ltd., Kent, UK). The coated samples were observed under SEM (FEI Quanta 600, FEI Company, Hillsboro, OR, USA) [30].

Energy dispersive spectroscopy (EDS) coupled with SEM was used for elemental analysis and was effective in locating and identifying NPs that were attached to the cells. Untreated cells and NPs attached to treated cells were spotted to analyze the elements. Three spots were selected in each sample to analyze the elements (Figures 4e, 5e, 6e, 7e, 8e and 9e).

Transmission electron microscopy (TEM) was employed to characterize the size of the NPs and to observe the morphology of the bacterial cells following treatment with ZnO and Ag NPs. Three samples of bacterial cells treated with and without NPs were fixed with a primary fixative and microwaved under vacuum conditions in a Pelco Biowave (Ted Pella, Inc., Redding, CA, USA) at 120 W. The samples were rinsed with 0.1 M cacodylate buffer and embedded in histogel, followed by a secondary microwave fixation with a buffered (0.1 M cacodylate, 0.01 M of 2-mercaptoethanol, and 0.13 M of sucrose) 1% osmium tetroxide. The samples were then quickly rinsed three times with 0.1 M 2-mercaptoethanol and 0.13 M of sucrose and then rinsed three times with ultrapure water. Then, samples were dehydrated with ethanol solutions (20, 50, 70, 90, and 100%) and 100% acetone solution. The samples were infiltrated with Spurr’s resin and polymerized at 60 °C for 24 h. The sample blocks were processed in 85 nm thin sections with Leica Ultracut UCT ultramicrotomes (Leica Microsystems GmbH, Wetzlar, Germany). The sections were placed on 200 mesh thin bar grids and post-stained for 20 min with 5% uranyl acetate and 10 min with Sato’s triple lead stain. Stained samples were then observed in a JEOL 1400 (JEOL, Ltd., Tokyo, Japan) transmission electron microscope [30].

### 2.5. Determination of Membrane Leakage

Overnight cultures of the three strains were inoculated into the respective broth medium containing 0, 12, 16, 20 mM of ZnO NPs and allowed to sit for 10 h at 37 °C. Similarly, strains were exposed to 0, 1.8, 2.7, 4.6 mM of Ag NPs for 6 h at 37 °C. After incubation, 1 mL of the treated bacterial suspensions was centrifuged at 18,200× *g* for 5 min and resuspended in sterile peptone water. The light absorbance of the suspensions was examined using a UV-visible spectrophotometer (UV-1650 PC, Suzhou Instruments Manufacturing Co. Ltd., Suzhou, China) at a wavelength of 260 nm (for DNA absorbance) and 280 nm (for protein absorbance). All experiments were replicated twice [30].

### 2.6. Assessment of the Viability of Bacterial Cells

To determine the viability of the treated cells, 1 mL of samples was centrifuged at 18,200× *g* for 5 min. Cell pellets were washed with 1 mL of 0.85% NaCl and stained using the *Bac*Light™ Bacterial Viability Kit (Invitrogen, Carlsbad, CA, USA) according to the manufacturer’s instructions. The samples were incubated at room temperature in the dark for 15 min and 8 µL of the stained bacterial suspension were placed between a glass slide and a 170 µm thick coverslip. Samples were observed under a Zeiss LSM 510 META (Zeiss LSM 510 META NLO, Carl Zeiss Ltd., Jena, Germany) confocal microscope [30].

### 2.7. Statistical Data Analysis

The SAS GLM procedure (SAS 9.2, Copyright 2002–2007; SAS Institute Inc., Cary, NC, USA) was used to evaluate the effects of ZnO and Ag NPs on growth and membrane leakage of bacterial strains. Tukey’s test was applied to determine differences between different concentrations of NP treatments at a significance level of 0.05.

## 3. Results 

### 3.1. Characterization of ZnO and Ag NPs

**Figure 1:**
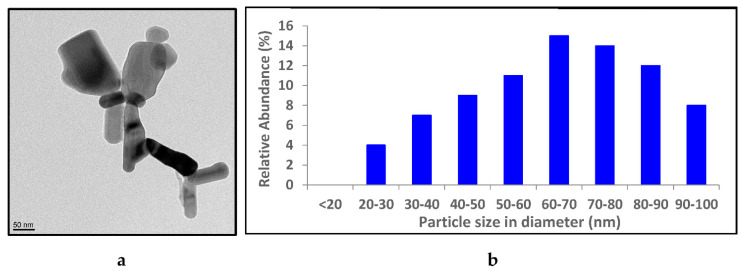

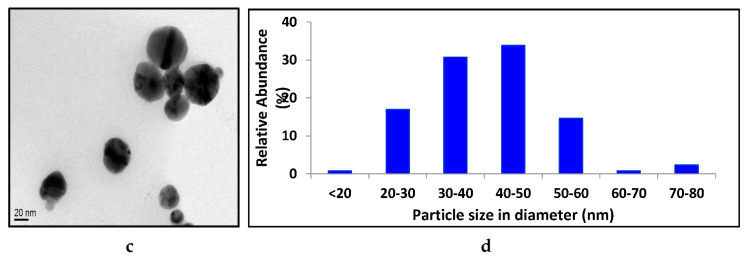
TEM image and size distribution of ZnO NPs (**a**,**b**) and TEM image and size distribution of Ag NPs (**c**,**d**).

Most of the ZnO NPs were either round or rectangular-shaped with an average size of 77.9 nm (Figure 1a). The size distribution histogram of the ZnO NPs indicated that the majority of the NPs were in the range of 60–80 nm in diameter (Figure 1b). According to Zhang et al. (2007), ZnO NPs may be present in the form of agglomerates due to synthetic processing. Hence, ultrasonication and dispersants, such as polyethylene glycol (PEG), polyvinylpyrolidone (PVP) and bovine serum albumin (BSA) are often used to disintegrate NP agglomerates [21]. However, the ZnO NPs used in this study were relatively well-dispersed with only slight agglomeration in ultra-purified water without the use of sonification or dispersants (Figure 1a). Also, incubating the cells with shaking allowed for the avoidance of ZnO NP aggregation in the broth.

The shape of Ag NPs observed under TEM was uniformly spherical and they were well-dispersed (Figure 1c). The majority of the Ag NPs measured fell in the range of 30–50 nm in diameter with an average size of 40.2 nm (Figure 1d) [30].

### 3.2. Effect of ZnO NPs on Bacterial Growth 

**Figure 2:**
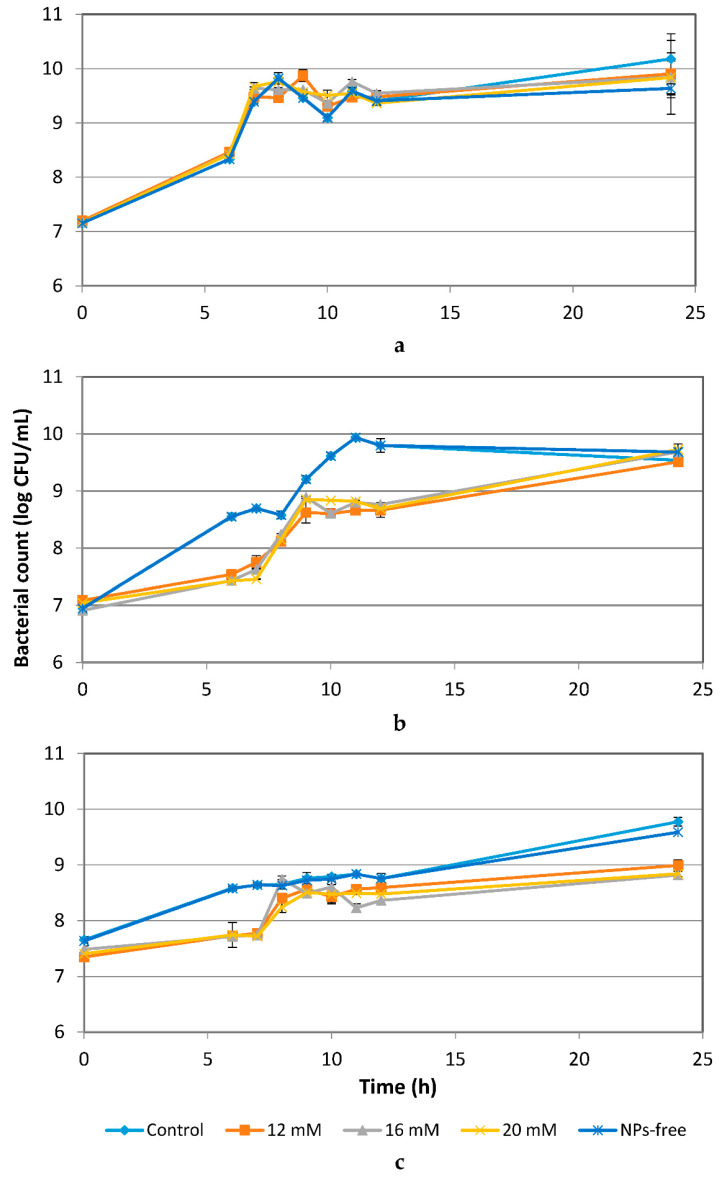
Effects of ZnO NPs on the growth of *E. coli* (**a**), *L. acidophilus* (**b**) and *B. animals* (**c**) over a 24-h period.

As shown in Figure 2, the growth of *E. coli* for the control and all treated samples, including the NP-free sample, exhibited very similar patterns. Even at the highest concentration (20 mM) of ZnO NPs, no significant effects on *E. coli* (*p* ≤ 0.05) were observed (Figure 2a). For *L. acidophilus*, the numbers of treated cells were within 1 log CFU/mL less than those of the control for up to 12 h of incubation. After 12 h, the cell numbers of treated samples increased slowly, and by the end of 24 h, their numbers showed no significant differences (*p* ≤ 0.05) as compared to the control (Figure 2b). Numbers of *B. animalis* in treated samples were less than 1 log CFU/mL lower than the control for up to 7 h of incubation. After 8 h, the number of treated cells followed similar patterns as the control and NP-free sample, and by the end of 24 h, the numbers of treated cells were within 1 log CFU/mL that of the control (Figure 2c). *L. acidophilus* was reduced by 11.4% after 10 h of exposure to ZnO NPs, which was the highest reduction percentage as compared to the other exposure times. About a 10% reduction of *B. animalis* numbers was observed between 6 and 7 h of exposure to ZnO NPs, after which, less than 5% of reduction was observed. However, at the end of 24 h, a more than 10% reduction in the number of *B. animalis* cells was observed (Figure 2c) [30].

### 3.3. Effect of Ag NPs on the Bacterial Growth

**Figure 3:**
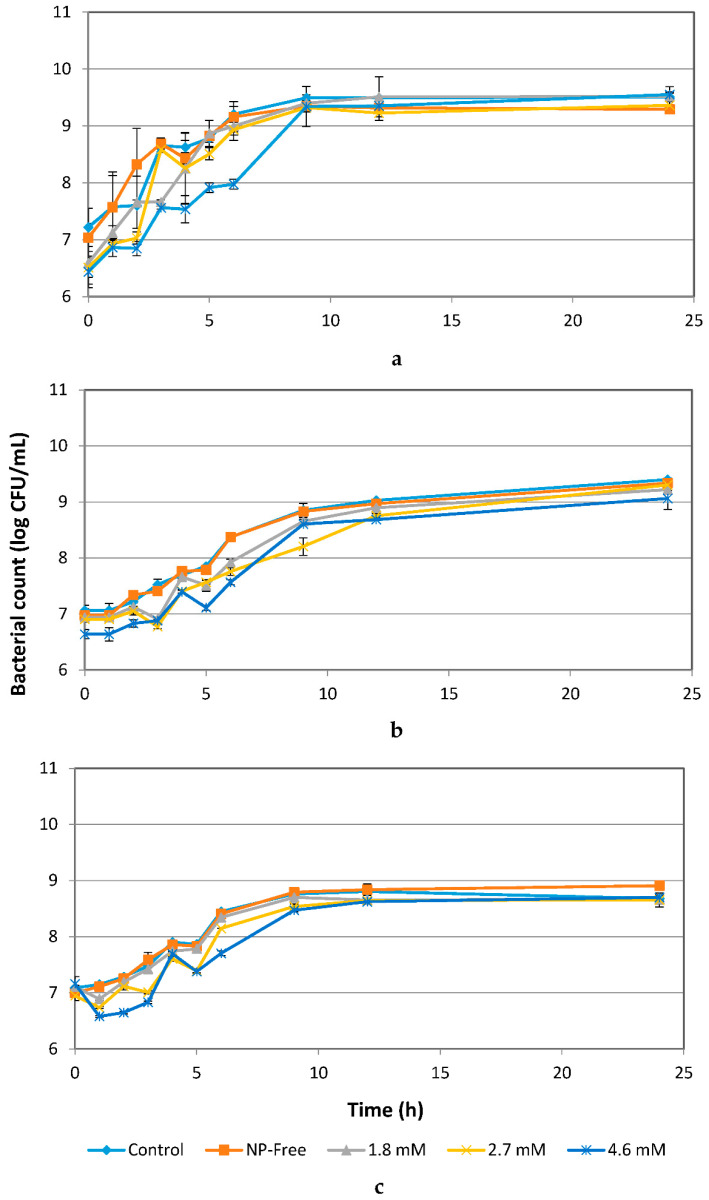
Effects of Ag NPs on the growth of *E. coli* *(***a**), *L. acidophilus* (**b**) and *B. animals* (**c**) over a 24-h period.

Ag NPs at 1.8, 2.7 and 4.6 mM had a significant inhibitory effect (*p* ≤ 0.05) on *E. coli* for up to 9 h (Figure 3a). Increasing concentrations of Ag NPs from 1.8 to 4.6 mM more greatly inhibited the growth of *E. coli* for up to 10 h, but no differences in numbers were seen as compared to the controls after 10 h. Compared to *E. coli*, *L. acidophilus* and *B. animalis* were less inhibited by Ag NPs (Figure 2b,c). The growth patterns of treated *L. acidophilus* and *B. animalis* were similar to those of their controls and NP-free controls. Concentrations of Ag NPs of up to 1.8 mM showed no significant effects on *L. acidophilus* and *B*. *animalis*. Ag NPs at 2.7 and 4.6 mM showed a significant effect (*p* ≤ 0.05) on *L. acidophilus* and *B*. *animalis* for up to 9 h. No significant effects of Ag NPs after 12 h of exposure were observed for *L. acidophilus* and *B*. *animalis*. After 9 h of exposure to Ag NPs, a less than 5% of cell reduction was observed [30].

### 3.4. Morphological Examination of Bacterial Cells Treated with ZnO and Ag NPs 

**Figure 4:**
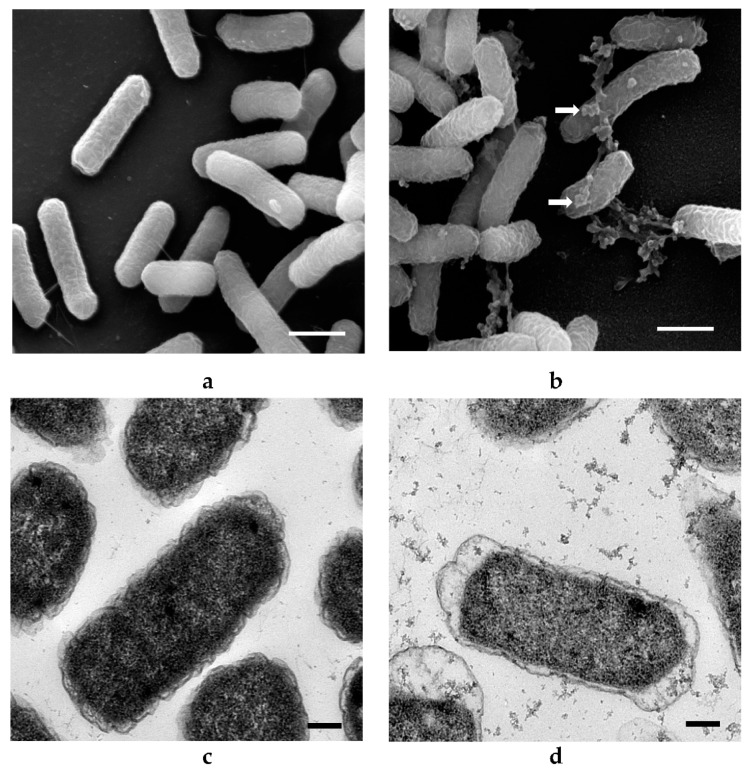

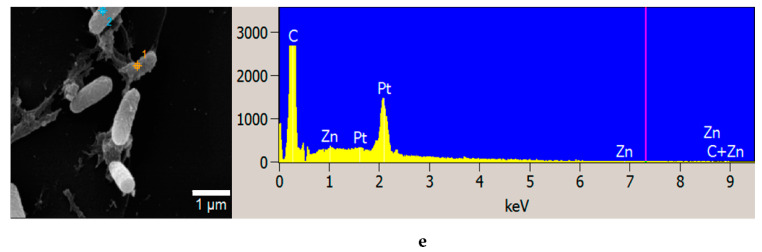
SEM (**a**,**b**) and TEM (**c**,**d**) images of *E. coli* untreated with ZnO NPs (**a**,**c**) and treated with 20 mM ZnO NPs (**b**,**d**). White arrows indicated clusters of ZnO NPs. SEM-EDS analysis shows ZnO presence on cell surfaces (**e**). SEM scale bar (**a**,**b**) = 1 μm, TEM scale bar (**c**,**d**) = 0.2 μm.

No significant changes in bacterial morphology (size, shape, appearance) were observed after ZnO NP treatments for 10 h (Figure 4). Some ZnO NPs were observed to adhere to *E. coli* cells (arrows, Figure 4b). The sizes of ZnO NPs attached to the bacterial cells appeared larger and clustered as compared to those demonstrated in the corresponding TEM image (Figure 4a). This may be due to agglomeration of ZnO NPs during the incubation period. TEM images allowed for direct visualization of intracellular morphological changes of bacterial cells before and after treatment with ZnO NPs. The bacterial cells were normal in size with intact intracellular structures and well-maintained intracellular contents (Figure 4c). Some deformations of bacterial cells were observed after treatment with ZnO NPs (Figure 4d). These include a distortion of the cell wall and cell membrane with overall cell shrinkage and a less dense appearance of the cytoplasm that may indicate membrane disruption and leakage of cell content when cells were treated with the higher concentration (20 mM) of ZnO NPs. However, not all cells were damaged, which supports the result of the growth studies where no effect of ZnO NPs on *E. coli* was demonstrated (Figure 2a) [30].

**Figure 5:**
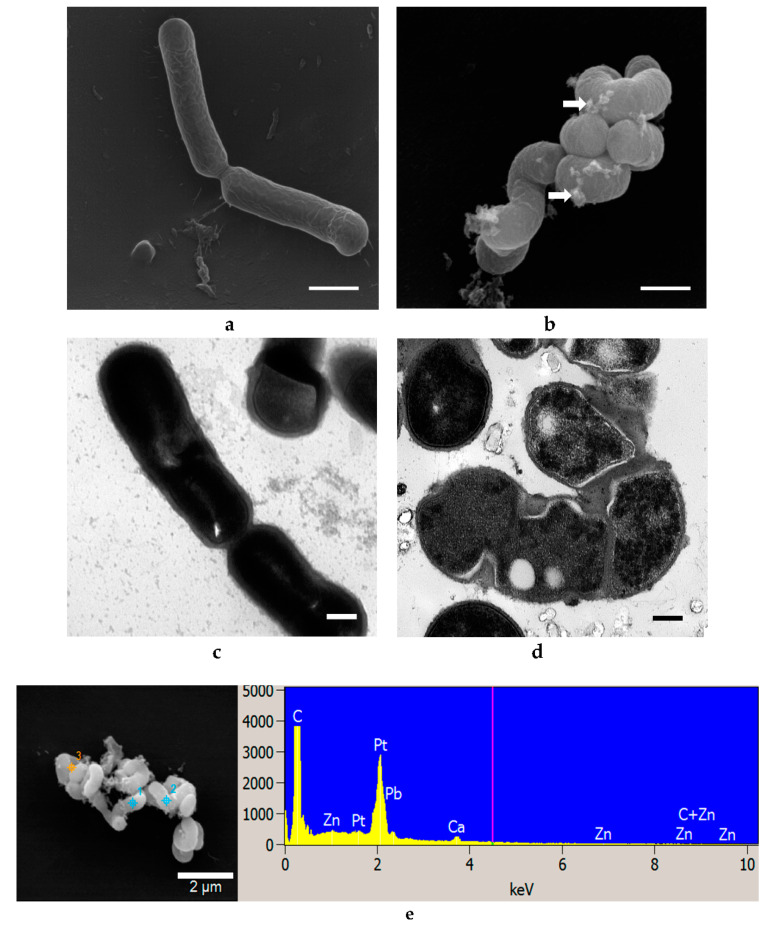
SEM (**a**,**b**) and TEM (**c**,**d**) images of *L. acidophilus* untreated with ZnO NPs (**a**,**c**) and treated with 20 mM ZnO NPs (**b**,**d**). SEM-EDS analysis shows ZnO presence on cell surfaces (**e**). White arrows indicated clusters of ZnO NPs. SEM scale bar (**a**,**b**) = 1 μm, TEM scale bar (**c**,**d**) = 0.2 μm.

**Figure 6:**
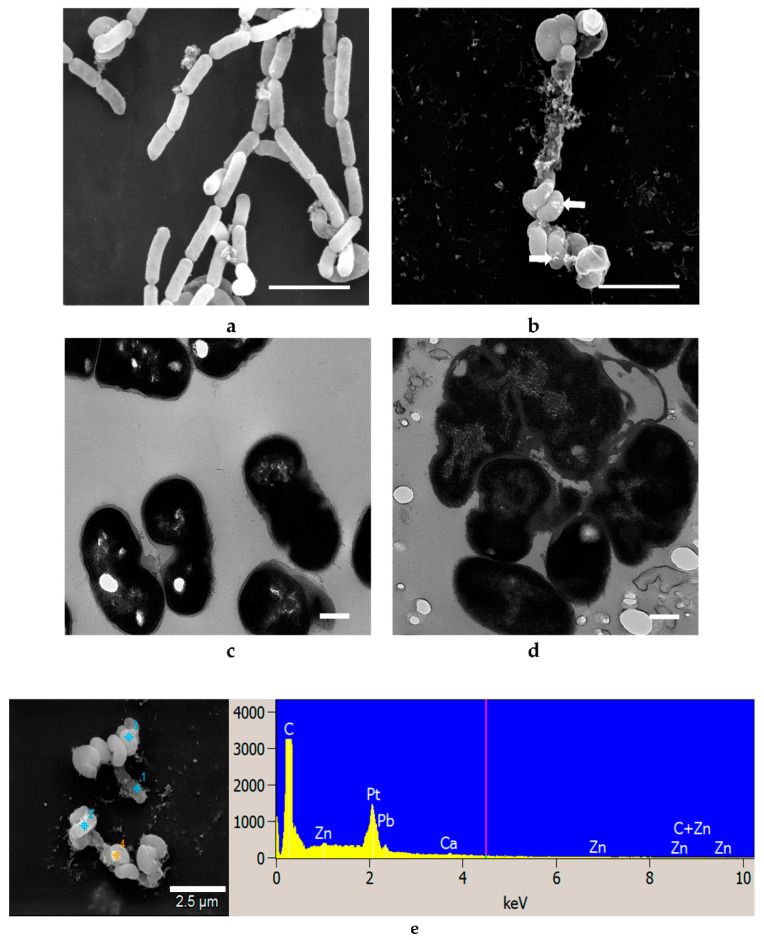
SEM (**a**,**b**) and TEM (**c**,**d**) images of *B. animalis* untreated with ZnO NPs (**a**,**c**) and treated with 20 mM ZnO NPs (**b**,**d**). SEM-EDS analysis shows ZnO presence on cell surfaces (**e**). White arrows indicated clusters of ZnO NPs. SEM scale bar (**a**,**b**) = 3 μm, TEM scale bar (**c**,**d**) = 0.2 μm.

Obvious changes in the cell morphology of *L. acidophilus* were observed after treatment with ZnO NPs, whereby ZnO NPs adhered to the cells (arrows, Figure 5b), and deformed them into a spiral and twisted shape. Many spiral or twisted shaped cells were found in the sample treated with ZnO NPs and TEM images show deformed cell membrane and intracellular morphological changes with cell leakage occurring in treated *L. acidophilus* (Figure 5d), as opposed to undamaged cells with a normal rod shape in the control group (Figure 5a,c). *B. animalis* showed similar morphological changes as *L. acidophilus* (Figure 6). Many deformed cells and spiral-shaped cells were observed in the treated samples. Further, damaged cells in the treated samples showed leakage of cell contents (Figure 6d) [30]. 

**Figure 7:**
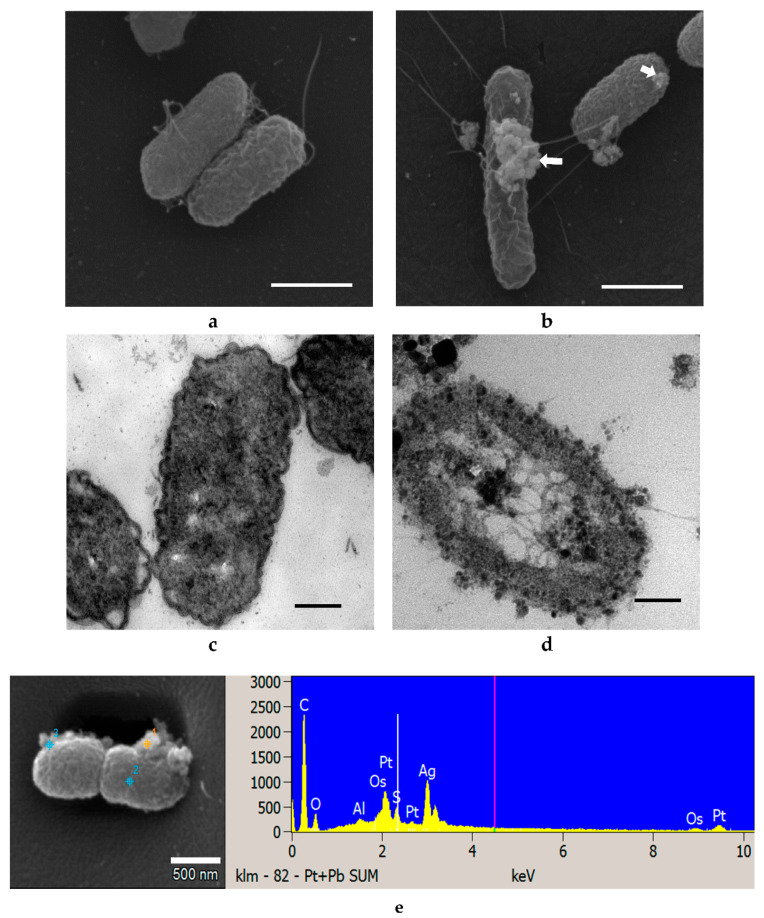
SEM (**a**,**b**) and TEM (**c**,**d**) images of *E. coli* untreated with Ag NPs (**a**,**c**) and treated with 4.6 mM Ag NPs (**b**,**d**). SEM-EDS analysis shows Ag NP presence on cell surfaces (**e**). White arrows indicated clusters of Ag NPs. SEM scale bar (**a**,**b**) = 1 μm, TEM scale bar (**c**,**d**) = 0.2 μm.

No significant changes in the external appearance of *E. coli* cells were observed after treatment with Ag NPs for 6 h (Figure 7). Ag NPs were seen attached to the bacterial cells (arrows, Figure 7b) in clusters instead of singly. Similar agglomeration was observed for ZnO NP-treated cells (Figure 4b and Figure 5b). Untreated cells were normal in size with intact intracellular structures and well-organized intracellular contents (Figure 7a,c). However, deformation of the cell membrane and an almost emptied cytoplasmic content was observed after treatment with 4.6 mM Ag NPs (Figure 7d). The bacterial cells were surrounded by Ag NPs and null cells found in samples treated with Ag NPs, indicating that intracellular contents had leaked due to damage to the cell membrane [30].

**Figure 8:**
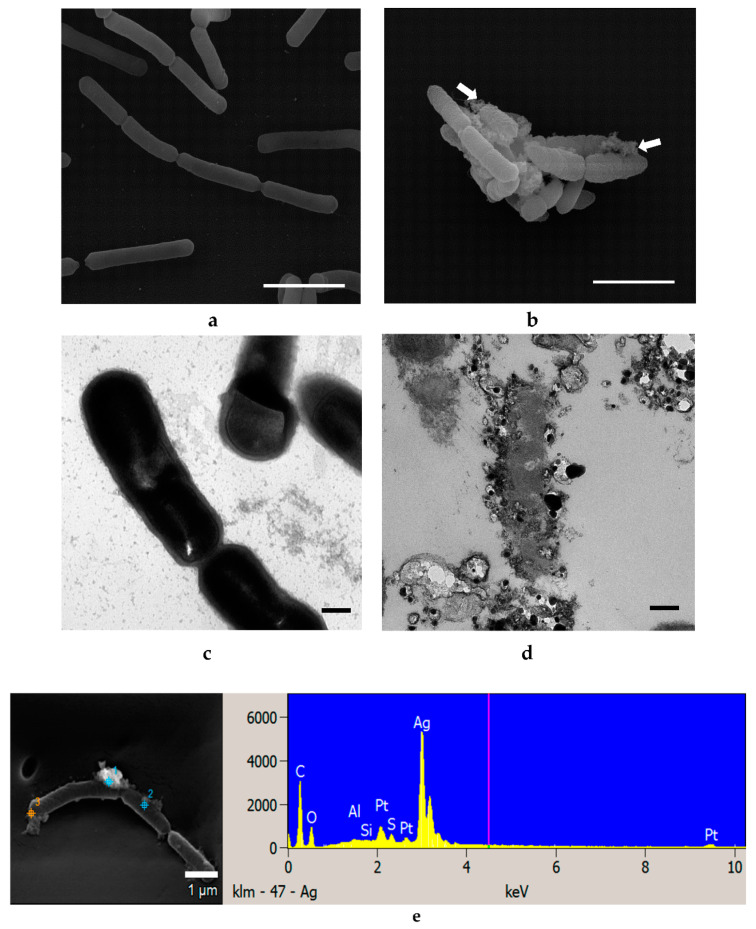
SEM (**a**,**b**) and TEM (**c**,**d**) images of *L. acidophilus* untreated with Ag NPs (**a**,**c**) and treated with 4.6 mM Ag NPs (**b**,**d**). SEM-EDS analysis shows Ag NP presence on cell surfaces (**e**). White arrows indicated clusters of Ag NPs. SEM scale bar (**a**,**b**) = 3 μm, TEM scale bar (**c**,**d**) = 0.2 μm.

**Figure 9:**
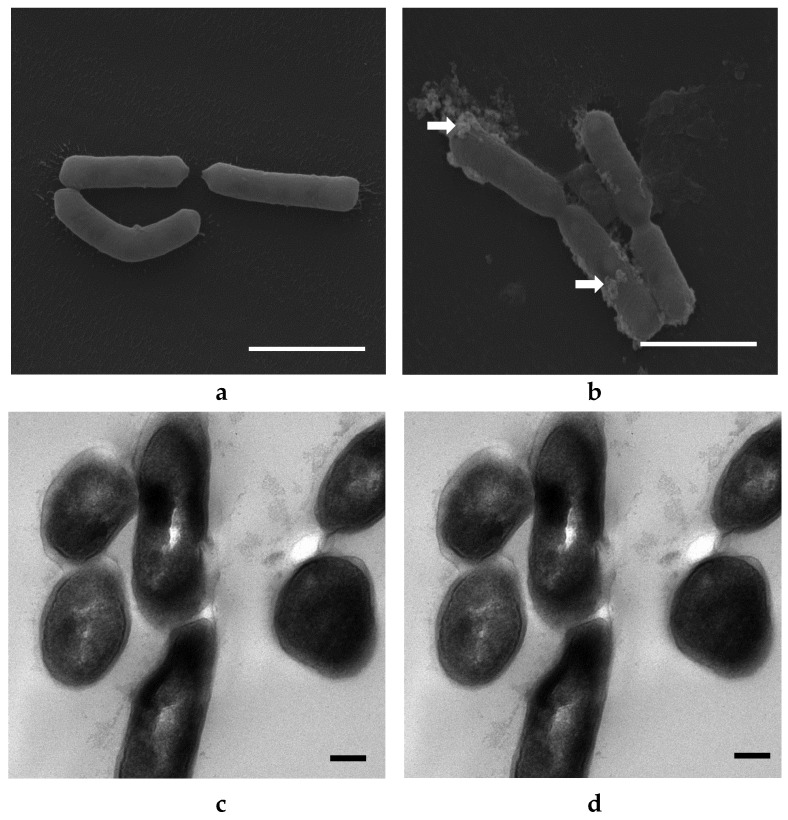

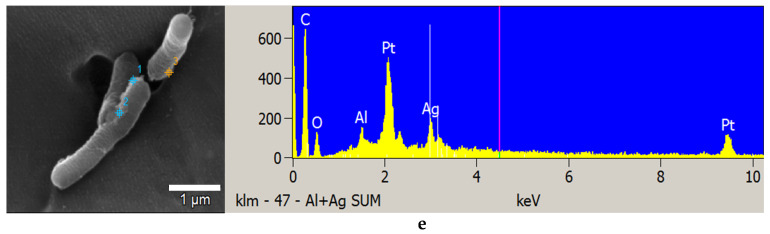
SEM (**a**,**b**) and TEM (**c**,**d**) images of *B. animalis* untreated with Ag NPs (**a**,**c**) and treated with 4.6 mM Ag NPs (**b**,**d**). SEM-EDS analysis shows Ag NP presence on cell surfaces (**e**). White arrows indicate clusters of Ag NPs. SEM scale bar = 2 μm, TEM scale bar = 0.2 μm.

No significant changes in bacterial morphology were observed before and after treatment with Ag NPs for *L. acidophilus* cells when observed under SEM (Figure 8a,b). However, TEM images of *L. acidophilus* showed some differences, such as distortion of cell membranes and leakage of internal contents of treated cells (Figure 8d). As seen in the SEM image (Figure 8b), not all cells were damaged, and there were still many cells in normal size and shapes. This is supported by the plate count number of about 10^7^ CFU/mL (Figure 3b), which is only 1 log CFU/mL less than the control. Similar results were observed with *B. animalis* (Figure 9) [30].

EDS is a common technique for analysis of the elemental composition of a specimen. It is also capable of generating a map of multiple chemical elements of interest at specifically pointed spots. SEM-EDS results show that no Zn or Ag elements were identified in the control cells of all three strains tested with either NPs but both elements were found on all respectively treated cells (Figure 4e, Figure 5e, Figure 6e, Figure 7e, Figure 8e and Figure 9e).

### 3.5. Determination of Membrane Leakage

**Figure 10:**
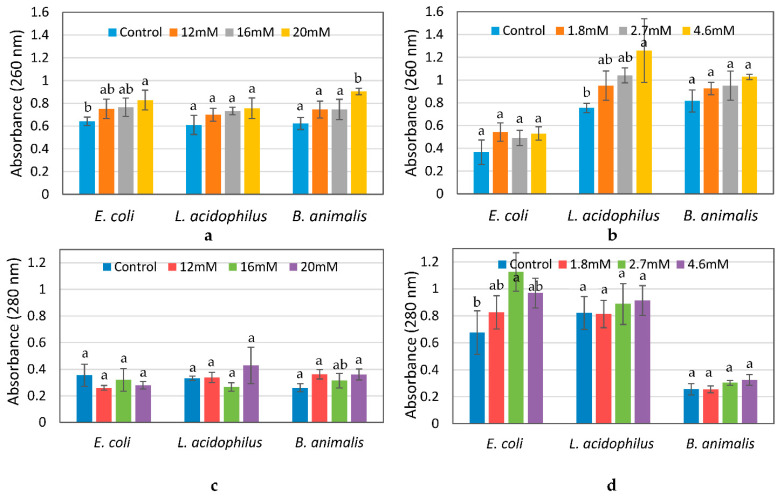
Nucleic acid (**a**,**b**) and protein (**c**,**d**) leakage from *E. coli*, *L.* *acidophilus*, and *B. animalis* after treatment with ZnO NPs (**a**,**c**) for 10 h and Ag NPs (**b**,**d**) for 6 h, as measured by absorbance at 260 nm and 280 nm, respectively. Different lowercase letters (a, b) indicate significant differences (*p* ≤ 0.05).

For all three bacteria, the absorbance at 260 nm slightly increased after 10 h of exposure to ZnO NPs and 6 h of exposure to Ag NPs as compared to the controls, which may be attributed to leakage of nucleic acids from cells whose membranes were damaged. However, there were no significant differences (*p* ≤ 0.05) in absorbance between the control and treated samples, except for *B. animalis* treated with the highest concentration of 20 mM ZnO and *L. acidophilus* treated with the highest concentration of 4.6 nM Ag NPs (Figure 10a,b). A similar trend was observed for protein leakage (Figure 10c,d) [30].

### 3.6. Viability of Bacterial Cells

**Figure 11:**
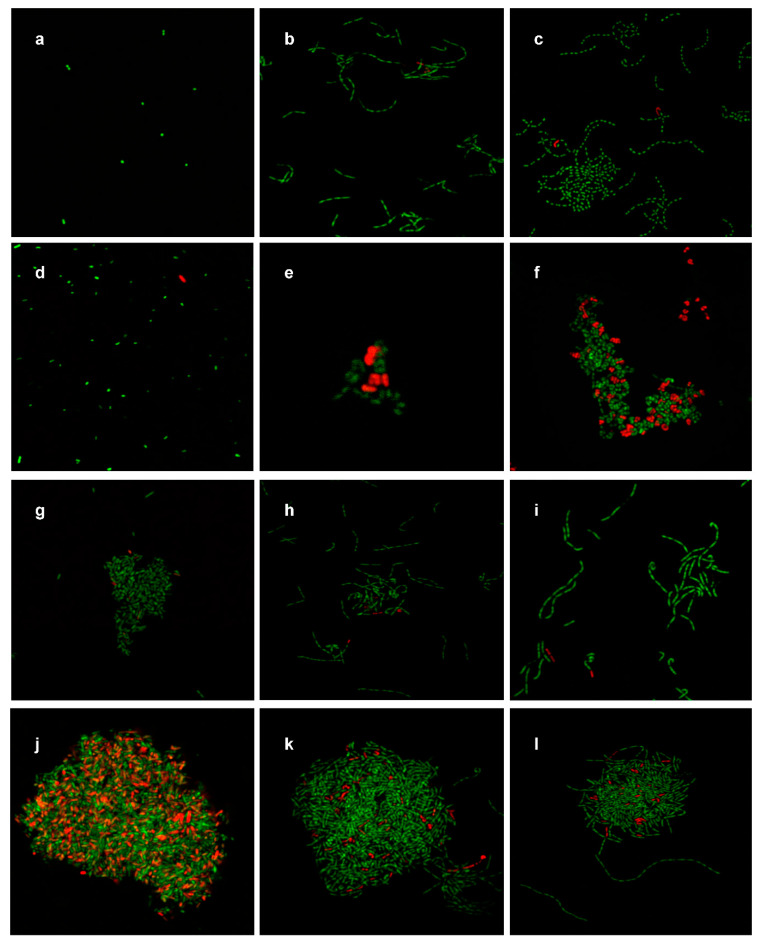
*Bac*Light™ fluorescence microscopic images of untreated *E. coli* (**a**,**g**), *L. acidophilus* (**b**,**h**) and *B. animalis* (**c**,**i**); 20 mM ZnO NP-treated *E. coli* (**d**), *L. acidophilus* (**e**) and *B. animalis* (**f**); and 4.6 mM Ag NP-treated *E. coli* (**j**), *L. acidophilus* (**k**) and *B. animalis* (**l**). Green indicates live cells while red indicates dead cells.

Images of *E. coli* control and ZnO NP-treated samples demonstrate mostly green fluorescence which indicated the presence of live cells (Figure 11a,d). Very few red fluorescent cells, indicating dead cells, were seen in the treated sample (Figure 11d), which supports the growth study results (Figure 1a) that demonstrated no significant effects (*p* ≤ 0.05) of ZnO NPs on *E. coli* growth, and the SEM/TEM images as shown on Figure 4. *L. acidophilus* and *B. animalis* showed similar morphological changes as those observed in the SEM/TEM images (Figure 5 and Figure 6) after treatment with ZnO NPs. Green fluorescent cells were observed in the control images with straight rod-shaped cells formed in a chain while, treated cells were formed in clusters or twisted around one another. However, not all twisted cells showed red fluorescence, which indicates that not all deformed cells were dead (Figure 9e and Figure 11e). The results support the plate count numbers shown in Figure 2b,c), that showed about 10^8^ CFU/mL of bacterial cells. This indicates that 20 mM ZnO NPs did not result in leakage of internal contents and death of all *L. acidiphilus* and *B. animalis* cells.

For cells treated with 4.6 mM Ag NPs, many red fluorescent cells, indicating dead cells, were observed, especially for *E. coli* samples (Figure 11j) as compared to the untreated control (Figure 11g), which supported the results from the growth study (Figure 2a) and the SEM/TEM images (Figure 7). Overall, a higher number of dead *E. coli* cells were observed following Ag NP treatment as compared to ZnO NP treatment. Mostly green fluorescent cells with straight rod shapes were observed in the untreated sample of *L. acidophilus* and *B. animalis* (Figure 11h,i), and both green and red fluorescent cells, in large clusters, were observed in the treated samples (Figure 11k,l). However, as compared to the ZnO NP treatment, more green fluorescent cells than red were observed in the treated samples, which indicated that more live cells of *L. acidophilus* and *B. animalis* were present. Thus, the results of the viability of cells show that not all cells were equally affected by ZnO or Ag NPs and resulted in cell death [30].

## 4. Discussion

In this study, the effects of ZnO and Ag NPs on the intestinal bacteria, *E. coli*, *L. acidophilus* and *B. animalis* were investigated. Compared to the antimicrobial effects of these NPs on pathogenic microorganisms demonstrated in previous studies [5,7,9,11,21,22,23,24,25,26,27,28,29,32,33,34,35,36,37,38,39], not much is known about the behavior of these NPs upon ingestion and whether they inhibit natural gut microflora. Antimicrobial properties of NPs have been shown to be dependent on their size and concentration, with increasing cytotoxicity at smaller sizes and higher concentrations [9,10,12,13,14,15,23,24,40].

The results in Figure 2b,c showed that the number of *L. acidophilus* and *B. animalis* treated with ZnO NPs was less than the control for up to 7–12 h of incubation, but subsequently followed similar patterns as the control and NP-free sample. This result is supported by a previous study that showed that, in addition to particle size and concentration of NPs, the antibacterial activity of NPs is also exposure time-dependent [24]. The results in Figure 2 indicated that concentrations of ZnO NPs higher than 12 mM showed mild inhibitory effects on the growth of the two Gram-positive bacteria, *L. acidophilus* and *B. animalis*, and no inhibitory effects on the growth of the Gram-negative *E. coli.* Similar results were observed in a study by Baek and An (2011) [32] in which *Staphylococcus aureus* and *Bacillus subtilis* (Gram-positive) were more susceptible than *E. coli* (Gram-negative) to NiO and ZnO NPs. The biosorption of metal NPs to bacterial cells depends not only on the types of NPs, but also on the microbial species [41]. A possible mechanism of antimicrobial effects of ZnO NPs has been suggested in several studies [8,9,42] as being generally triggered by the induction of oxidative stress by free radical formation that results in cell death. According to Pan et al. (2009) [43], ZnO NPs have weak mutagenic properties that induce frameshift mutations in *Salmonella* Typhimurium. However, there are many reports on the antibacterial effect of NPs that contradict one another which indicate that the inhibitory mechanisms of antimicrobial agents are very complicated and depend on many factors. Also, there are several bacteria that are present in the environment that naturally adapt to NPs and become tolerant to these compounds [44]. Hence, there is also a possibility that this might be the reason for our observations of the lack of inhibitory effects of ZnO NPs on *E. coli* in this study.

The results shown in Figure 3 indicate that Ag NPs had a significant inhibitory effect (*p* ≤ 0.05) on *E. coli* but less so on *L. acidophilus* and *B. animalis*. The mechanisms of the inhibitory effects of Ag NPs on microorganisms are partially known. Some studies have reported that the positive charge on the Ag^+^ ion is crucial for its antimicrobial activity through electrostatic attractions between the negatively-charged cell membrane of microorganisms and positively-charged Ag NPs [33,34,37,45]. However, there is almost no Ag^+^ ion release under anaerobic conditions, indicating that oxygen dissolved in water is necessary to convert Ag NPs to Ag^+^ ions [38,46]. Because they are both facultative anaerobes, *L. acidophilus* and *B. animalis* were incubated in anaerobic conditions. Hence, there was not enough oxygen available for the Ag NPs to release Ag^+^ ions. This could explain why these two bacteria were less inhibited by Ag NPs as compared to *E. coli.* Without oxygen, Ag NPs cannot release Ag^+^ ions which would have affected the growth of the bacterial cells. The slight inhibitory effect on the growth of *L. acidophilus* and *B. animalis* may be due to the exposure of the Ag NPs to oxygen during preparation and experiments that resulted in the release of minimal numbers of Ag^+^ ions. Additionally, compared to Ag NPs, ZnO NPs showed higher inhibitory effects on *L.* acidophilus and *B. animalis.* This is because ZnO NPs can release Zn^+^ ions regardless of the presence of oxygen. Another possible mechanism, as described by Amro et al. (2000) [47], is that metal depletion may cause the formation of irregularly-shaped pits on the outer membrane of cells that can change the membrane permeability and cause leakage of internal cell contents. However, the exact mechanisms of the antibacterial property of NPs to different types of bacteria are still not completely understood.

Unlike the results of ZnO NPs on *E. coli*, which showed higher antimicrobial effects than Ag NPs to *L. acidophilus* and *B. animalis*, other studies showed similar results as those observed in this study. *E. coli* O157:H7 cells were inhibited by Ag NPs at lower concentrations as compared to *S. aureus* [11]. Also, the minimum inhibitory concentration of Ag NPs was lower when testing against *E. coli* than when testing against *S. aureus* [24]. However, there is no supporting evidence to explain the species sensitivity to NPs.

As shown in Figure 4, Figure 5, Figure 6, Figure 7, Figure 8 and Figure 9, compared to cells without NP treatment which showed well-organized single cells and non-clustered cells, cells treated with ZnO or Ag NPs agglomerated together and formed large clusters. A possible explanation may be electrostatic attractions between the negatively-charged cell membrane of the cells and the positively-charged Zn^+^ or Ag^+^ ions from NPs. Further, although *L. acidophilus* and *B. animalis* were incubated in anaerobic conditions, similar agglomerations of both NPs were observed. This may be due to the exposure of Ag NPs to oxygen and the release of Ag^+^ ions during preparation of the bacterial viability assay and fluorescence microscopy that subsequently reacted with the negatively-charged bacterial surfaces.

In summary, our results showed that bacterial strains exposed to ZnO NPs for 10 h and Ag NPs for 6 h assumed the most antimicrobial effect. However, the number of treated cells were within 1 log CFU/mL less than that of the control and the reduction percentage in the number of cells was about 10% or less. The results of SEM and TEM images and EDS demonstrated the morphological changes of the cells and the adherence of the NPs to bacterial cells. Some externally and internally damaged cells were observed. However, not all cells were damaged, and there were still many cells in normal size with intact intracellular structures and well-organized intracellular contents which correlated with the results of cell reduction. Also, UV absorbance values indicated that no significant amounts of internal cellular contents were leaked due to NP exposures (Figure 10). Finally, the viability assay of bacterial cells confirmed that more live than dead cells were present after treatment with NPs. The overall results indicate that not all cells were affected by NPs (Figure 11).

## 5. Conclusions

In this study, the antimicrobial effects of ZnO and Ag NPs on three important intestinal bacteria were determined because of the potential exposure of intestinal flora to these nanoparticles. According to the results obtained in this study, it can be concluded that ZnO and Ag NPs have mild inhibitory effects on the three common intestinal bacteria tested. It should be noted that the contamination level of ZnO and Ag NPs in food samples, if present, would be very low, and much higher concentrations of NPs were tested in this study. The data obtained from this study indicate that food contaminated with ZnO and Ag NPs may offer only a negligible threat to the specific beneficial gut bacteria tested. However, mechanisms of antimicrobial effects of NPs are not yet fully understood. Studies in this area have reported various mechanisms of action of NPs, most widely reported being the generation of reactive oxygen species that subsequently lead to cellular disruptions [48,49]. Data from one study may not be comparable to those of others due to differences in sizes of NPs tested, manner in which they have been generated, and conditions of exposure of the NPs to the cells being tested. Continued studies on the mechanisms of antibacterial effects of ZnO and Ag NPs are clearly needed. In addition, there are limitations, such as stability, bioaccumulation, and toxicity features when synthesizing NPs using chemical vapor deposition, sol-gel process, spray pyrolysis, laser pyrolysis, and molecular condensation methods [50,51]. Recently, the green synthesis method offers a novel and potential alternative way to chemically-synthesized NPs [16,17,18,19]. For further studies, investigations into the antimicrobial effects of NPs synthesized by green synthesis methods on various kinds of bacteria would be worthwhile.

## Data Availability

The data presented in this study are available by request to the corresponding author.
